# Detection of anthracycline-induced cardiotoxicity using perfusion-corrected ^99m^Tc sestamibi SPECT

**DOI:** 10.1038/s41598-018-36721-5

**Published:** 2019-01-18

**Authors:** Zaitulhusna M. Safee, Friedrich Baark, Edward C. T. Waters, Mattia Veronese, Victoria R. Pell, James E. Clark, Filipa Mota, Lefteris Livieratos, Thomas R. Eykyn, Philip J. Blower, Richard Southworth

**Affiliations:** 1 School of Biomedical Engineering and Imaging Sciences, London, UK; 2 Department of Neuroimaging, London, UK; 3 School of Cardiovascular Medicine and Sciences, London, UK

## Abstract

By the time cardiotoxicity-associated cardiac dysfunction is detectable by echocardiography it is often beyond meaningful intervention. ^99m^Tc-sestamibi is used clinically to image cardiac perfusion by single photon emission computed tomography (SPECT) imaging, but as a lipophilic cation its distribution is also governed by mitochondrial membrane potential (ΔΨ_m_). Correcting scans for variations in perfusion (using a ΔΨ_m_-independent perfusion tracer such as (bis(N-ethoxy-N-ethyldithiocarbamato)nitrido ^99m^Tc(V)) (^99m^Tc-NOET) could allow ^99m^Tc-sestamibi to be repurposed to specifically report on ΔΨ_m_ as a readout of evolving cardiotoxicity. Isolated rat hearts were perfused within a γ-detection apparatus to characterize the pharmacokinetics of ^99m^Tc-sestamibi and ^99m^Tc-NOET in response to mitochondrial perturbation by hypoxia, ionophore (CCCP) or doxorubicin. All interventions induced ^99m^Tc-sestamibi washout; hypoxia from 24.9 ± 2.6% ID to 0.4 ± 6.2%, CCCP from 22.8 ± 2.5% ID to −3.5 ± 3.1%, and doxorubicin from 23.0 ± 2.2% ID to 17.8 ± 0.7, p < 0.05. Cardiac ^99m^Tc-NOET retention (34.0 ± 8.0% ID) was unaffected in all cases. Translating to an i*n vivo* rat model, 2 weeks after bolus doxorubicin injection, there was a dose-dependent loss of cardiac ^99m^Tc-sestamibi retention (from 2.3 ± 0.3 to 0.9 ± 0.2 ID/g with 10 mg/kg (p < 0.05)), while ^99m^Tc-NOET retention (0.93 ± 0.16 ID/g) was unaffected. ^99m^Tc-NOET therefore traps in myocardium independently of the mitochondrial perturbations that induce ^99m^Tc-sestamibi washout, demonstrating proof-of-concept for an imaging approach to detect evolving cardiotoxicity.

## Introduction

Anthracyclines are a first in line therapy used to treat numerous malignancies including leukaemia, lymphoma, myeloma, lung, ovarian, gastric, thyroid and breast carcinoma^1,2^. However, they are highly toxic to the heart, inducing coronary heart disease, valvular heart disease, cardiomyopathy, and heart failure^3,4^. This is of particular concern for paediatric cancer patients, cardiovascular disease being the leading non-malignant cause of death in long-term survivors of childhood cancer^5^. Anthracycline-induced cardiac injury is generally dose-dependent^6^ and the severity and rate of progression of cardiotoxicity varies greatly between patients being dependent on age^7^, gender^8^, co-existing disease^7^, prior or concurrent chemo- or radiotherapy^9,10^, and rate and frequency of drug administration^11^. Personalizing chemotherapeutic dose while minimizing cardiac damage is highly challenging^12^, the timeline of cardiotoxicity development can vary anything from weeks to years following chemotherapy^2^, which is further confounded by an asymptomatic period prior to a progressive functional deterioration^13^.

Cardiotoxicity is routinely monitored by functional measurements using echocardiography or multigated acquisition (MUGA) scanning^14^. However, anthracycline cardiotoxicity is initiated at the subcellular level, commonly associated with mitochondrial dysfunction and elevated oxidative stress^15–17^. By the time cardiotoxicity manifests as contractile dysfunction, it is often too late to intervene beyond managing the heart failure^18^. Non-invasive *in vivo* molecular imaging approaches, targeted at mitochondrial dysfunction, may be able to highlight evolving cardiotoxicity earlier and with greater sensitivity than functional imaging approaches currently employed.

Fluorescent lipophilic cations like tetramethylrhodamine ethyl ester (TMRE) and Rhodamine123 are extensively used experimentally to report on mitochondrial membrane potential (ΔΨ_m_) in cell culture and isolated tissue preparations. However, they have limited utility *in vivo* due to poor depth penetration, and lack of whole body scanning. On the other hand, ^99m^Tc-sestamibi, (^99m^Tc-MIBI) is routinely used clinically to visualize cardiac perfusion using SPECT, but as a lipophilic cation^19^ its myocardial distribution is more correctly a function of both regional perfusion *and* ΔΨ_m_^20^. If cardiac retention of ^99m^Tc-MIBI or other SPECT and PET lipophilic cations could be corrected for perfusion, either by pharmacokinetic modelling or using a parallel “true” perfusion imaging agent which is independent of mitochondrial function or other metabolic parameters, it may be possible to develop tracers that report on cardiac ΔΨ_m_ as a non-invasive clinical readout of mitochondrial cardiotoxicity. Such an approach would provide more responsive, accurate, personalized treatment regimes, or could be employed as an imaging biomarker of novel cardioprotective regimes or therapies.

In this study, we investigated the possibility of pairing ^99m^Tc-MIBI with ^99m^Tc-NOET (bis(N-ethoxy-N-ethyldithiocarbamato)nitrido ^99m^Tc(V)), which is a lipophilic uncharged (and therefore ΔΨ_m_ independent) “true” perfusion imaging agent^21^ to report on cardiotoxicity by SPECT.

## Materials and Methods

### Reagents and gas mixtures

All reagents were purchased from Sigma Aldrich (Poole, Dorset, UK) unless otherwise stated. All gas mixtures were purchased from BOC, UK. Specialist gas mixtures were certified by the manufacturer.

### Animals

Male Wistar rats (220–240 g, B&K Universal, UK) were used for all experiments in accordance with the Animals (Scientific Procedures) Act, UK (1986). All experimental procedures were approved by King’s College London’s local Animal Care and Ethics Committee, and carried out in accordance with Home Office regulations as detailed in the Guidance on the Operation of Animals (Scientific Procedures) Act 1986.

### Radiopharmaceutical preparations

^99m^Tc-MIBI was prepared from commercial cold kits (Mallinckrodt Pharmaceuticals) reconstituted with saline (100MBq/ml) from a ^99^Mo/^99m^Tc generator and supplied by the radiopharmacy at Guy’s Hospital, UK. NOET ligand, DIMEB and Vial A were a generous gift from Roberto Pasqualini (co-inventor of ^99m^Tc-NOET)^22^. ^99m^Tc-NOET was prepared fresh before each use.

### The triple γ-detection system

We have described the design of our triple γ-detection system, and its use for pharmacokinetic characterization of radiotracers in the Langendorff perfused heart previously^23,24^. Briefly, it comprises three orthogonal lead-collimated Na/I γ-detectors arrayed around a perfusion apparatus, positioned (i) 3 cm downstream of a radiotracer injection port on the arterial line, 15 cm upstream of the heart cannula (to provide a radiotracer input function), (ii) directly opposite the heart (to monitor cardiac retention), and (iii) over the venous outflow line (to provide an output function). Each detector was connected to a modified GinaSTAR™ ITLC system running Gina™ software for real-time data collection (Raytest Ltd, UK).

### Langendorff Perfusion and radiometric analysis

Rats (n = 5/group) were anesthetized with Sagatal, (Rhone Merieux, 100 mg intraperitoneal), heparinised (200 IU intraperitoneal), and their hearts were excised and perfused in Langendorff mode with modified Krebs-Henseleit buffer (KHB) 37 °C containing NaCl (118.5 mM), NaHCO_3_ (25 mM), D-glucose (11 mM), KCl (8 mM), CaCl_2_ (2.5 mM), MgSO_4_ (1.2 mM), and Na2-ethylenediamine tetraacetic acid (0.5 mM) at 37 °C gassed with 95%O_2_/5%CO_2_ at 14 mL/min constant flow^23^, which on average delivered an initial coronary perfusion pressure of 74 mmHg (Supplemental Fig. 4). Buffer oxygen saturation was monitored by an arterial line fibre-optic oxygen/temperature probe (Oxford Optronix Ltd). Contractile function was monitored with an intraventricular balloon set to an initial end diastolic pressure of 6–8 mm Hg connected to a Powerlab system running Labchart software (AD Instruments Ltd).

Radiotracer boluses (4MBq in ~100 µl) were injected into the arterial line, and their passage through the triple γ-detector system evaluated. Cardiac tracer retention was calculated as the activity at 15 minutes post injection as a percentage of the peak cardiac activity immediately after each injection^23^. All curve fitting was performed using MATLAB (MathWorks®). Data were normalized to the maximum peak counts after each injection and corrected for decay and cardiac activity immediately prior to each bolus injection. Washout curves described by the temporal data f(t) were fitted to tri-exponential (Eq. 1) or bi-exponential functions (Eq. 2) as determined most appropriate using least squares fitting^24^:

1 $${\rm{f}}({\rm{t}})={{\rm{Ae}}}^{-{\rm{bt}}}+{{\rm{Ce}}}^{-{\rm{dt}}}+{{\rm{Fe}}}^{-{\rm{pt}}}$$

2 $${\rm{f}}({\rm{t}})={{\rm{Ae}}}^{-{\rm{bt}}}+{{\rm{Fe}}}^{-{\rm{pt}}}$$

where b, d and p were the fast, intermediate and slow clearance rate constants (FCR, ICR and SCR, respectively) and A, C and F were their respective weights.

### Experimental protocols

Langendorff experiments consisted of a 10-minute stabilization period, followed by a 30-minute control perfusion period, and then a 50-minute intervention period. Radiotracer boluses were injected after 10 minutes of control perfusion, and then 5 and 25 minutes after the induction of each intervention. The effect of cardiac hypoxia on tracer pharmacokinetics was investigated by switching perfusion buffer to a second reservoir containing KHB equilibrated with 95%N_2_/5% CO_2_. The effect of mitochondrial depolarization was investigated using the ionophore carbonyl cyanide m-chlorophenyl hydrazone (CCCP) at 0, 150, 300 and 600 nmol/L infused into the arterial line via a side arm. The direct acute effect of doxorubicin administration at 0, 1, 10 or 100 μmol/l was evaluated in the same manner. To determine our capacity to detect doxorubicin-induced cardiotoxicity *in vivo*, two additional studies were performed. Following baseline *in vivo* measurement of cardiac geometry and contractile function by echocardiography, rats were injected with doxorubicin (intraperitoneal; saline vehicle, 1, 5, or 10 mg/kg in 100 μl). Two weeks later, a follow-up echocardiographic assessment was performed. In the first study, hearts were excised and Langendorff-perfused as above. After a 20 min stabilization period, a bolus of either ^99m^Tc-MIBI or ^99m^Tc-NOET was injected into the arterial perfusion line, and cardiac retention and washout were determined using the triple γ-detection system as before. In the second study, animals were injected with either ^99m^Tc-MIBI or ^99m^Tc-NOET (100 MBq in 50 μl via tail vein). 3 hours later, animals were culled, and tracer biodistribution (including cardiac retention) was determined by a standard *ex vivo* cut-and-count approach. The apex of the myocardium was dissected *in situ*, from which 2 mm cubes of ventricular tissue were cut and immediately immersed in fixative (2.5% glutaraldehyde in 0.1 M sodium cacodylate buffer (pH 7.4)) for electron microscopy. The remainder of the myocardium was freeze-clamped with liquid nitrogen and stored at −80 °C prior to biochemical analysis.

### Echocardiography

Echocardiograms were obtained using a Vevo 770 System (VisualSonics, Canada) with a RMV-710B transducer running at 25 MHz. B-mode imaging was used to obtain high-resolution two-dimensional cross-sections in long- or short-axis view. M-Mode imaging then interrogated a line of interest at the level of a papillary muscle to measure the thickness of the interventricular septum in diastole (IVSs), left ventricular internal diameter in diastole (LVIDd), left ventricular internal diameter in systole (LVIDs) and left ventricular posterior wall thickness in diastole (LVPWd). These measurements were used to calculate left ventricular fractional shortening (FS) and ejection fraction (EF) in accordance with American Society for Echocardiography Guidelines.

### Electron microscopy

Samples were fixed, stained and sectioned as previously described^25^. Images were captured using a Fei Tecnai 12 transmission electron microscope at an accelerating voltage of 80 kV. To ensure that there was no bias in our measurements, we used “systematic random sampling” to obtain 60 images/heart, as previously described^26^. Mitochondrial area and number were calculated using our previously described stereological approach, while the percentage of damaged mitochondria in each field of view were counted and averaged across all sections.

### High energy phosphate assay

Cardiac high energy phosphate levels were assayed by reversed-phase HPLC according to Teerlink *et al*.^27^ using a 5 µm Eclipse XDB-C18 15 cm × 4.6 mm column (Agilent Technologies). ATP and ADP were quantified by measuring the UV peak area at 210 nm compared to corresponding standards, expressed as µmol/g wet tissue.

### Citrate Synthase Assay

Citrate synthase was measured spectrophotometrically as previously described^28^. 50 mg of tissue was homogenized in 1 ml STE buffer (250 mM sucrose, 5 mM Tris, 1 mM EDTA, pH 7.4) and centrifuged for 1 min at 6000 rpm, 4 °C. The supernatant was removed and protein concentration determined by BCA assay. Protein (20 μg) was incubated for 2 min in a 1 ml cuvette thermostatted to 30 °C containing 900 µl 100 mM Tris HCl, pH 8.0, 0.1% (v/v) Triton-X-100, 0.04 mg/ml DTNB, and 0.3 mg/ml acetyl CoA. Absorbance was measured at 412 nm every 2 seconds for 2 min on a SPECTROstar Nano spectrophotometer. Oxaloacetic acid (0.05 mg/ml) was added and change in absorbance followed for 3 min. Background acetyl CoA hydrolysis was corrected for by subtracting the rate prior to oxaloacetic acid addition from the rate post addition. Data were normalized to sample protein content and expressed as change in absorbance/min/μg protein. Each sample was run in triplicate and averaged.

### Data analysis

All group allocations and analyses were performed randomized and blinded, with the identities of the tissue samples and images revealed only after the data analysis was complete. Statistical analysis was performed using GraphPad Prism® (GraphPad, USA). All values are expressed as mean ± SD. All data were analyzed using a one-way ANOVA with Bonferroni correction post-hoc test or Dunnett’s test when multiple comparisons were made to a control group.

## Results

### Langendorff tracer retention in response to mitochondrial perturbation

#### Hypoxia

**Figure 1:**
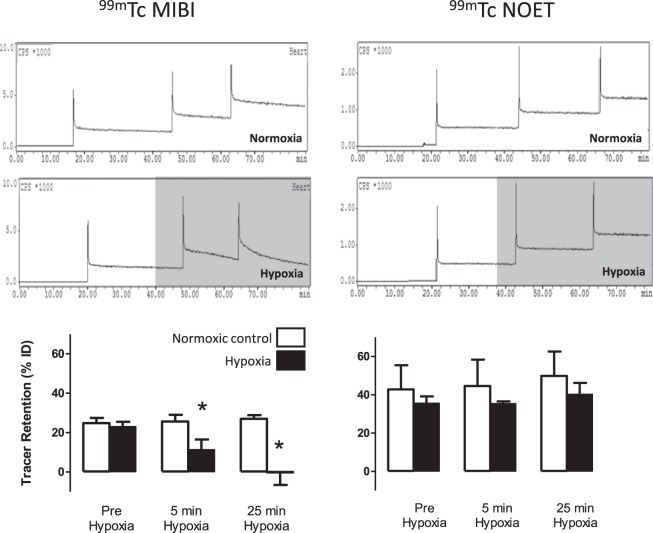
Time-activity curves showing the myocardial clearance/accumulation of ^99m^Tc-MIBI (left) and ^99m^Tc-NOET (right) during normoxic (top) and hypoxic buffer perfusion (middle). Graphs represent summarised data of mean (n = 6) ± SD. *Significantly different from normoxia (p < 0.05).

During normoxic perfusion, 24.9 ± 2.6% of each injected ^99m^Tc-MIBI dose was stably trapped in the heart (Fig. 1). Hypoxic buffer perfusion caused this trapping to fall to 11.3 ± 5.2% (p < 0.05) after 5 minutes and 0.4 ± 6.2% after 45 minutes (p < 0.05). ^99m^Tc-NOET was stably retained within the myocardium during aerobic perfusion (34 ± 8% of injected dose), and its retention was unaffected by hypoxic buffer perfusion. ^99m^Tc-MIBI time activity curves were best fitted by a tri-exponential equation. The FCR (16.9 ± 3.9 min^−1^) and its amplitude (0.65 ± 0.06) were unaffected by perfusion with hypoxic buffer (Supplementary Fig. 1). The ICR (1.67 ± 0.62 min^−1^) and its amplitude (0.08 ± 0.02) both approximately doubled with hypoxic buffer perfusion (p < 0.05), while the SCR (0.010 ± 0.002 min^−1^) increased 17-fold (p < 0.05). Its amplitude (0.27 ± 0.03) was not affected. ^99m^Tc-NOET kinetics were best fit with a bi-exponential equation. The initial FCR for ^99m^Tc-NOET was 30.05 ± 4.7 min^−1^ with an amplitude of 0.71 ± 0.13. This did not change significantly during continued normoxic or hypoxic buffer perfusion. The normoxic SCR was 0.003 ± 0.002 min^−1^, which approximately doubled during hypoxia (p < 0.05), while its amplitude (0.389 ± 0.128) did not change.

#### CCCP infusion

**Figure 2:**
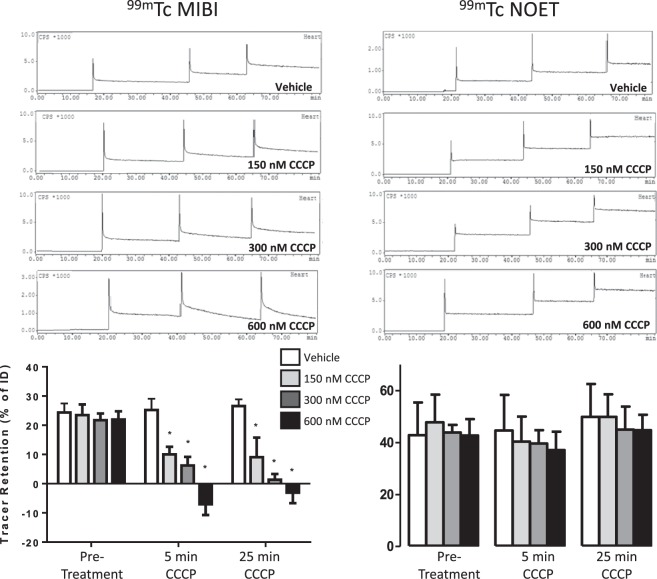
Time-activity curves showing the myocardial clearance/accumulation of ^99m^Tc-MIBI (left) and ^99m^Tc-NOET (right) during perfusion with increasing concentrations of CCCP. Graphs represent summarised data of mean (n = 6) ± SD. *Significantly different from pre-intervention control bolus (p < 0.05).

Increasing concentrations of CCCP caused a progressive loss of cardiac ^99m^Tc-MIBI trapping, falling from 22.8 ± 2.5% to 10.5 ± 2.2% and 6.7 ± 2.5% after 5 minutes of perfusion with 150 and 300 nM CCCP respectively (Fig. 2). After 5 minutes of perfusion with 600 nM CCCP perfusion, the cardiac capacity to trap ^99m^Tc-MIBI was compromised such that not only was the injected dose not retained, but tracer trapped within the heart from prior injections also washed out resulting in calculated retention values lower than baseline (−3.5 ± 3.1% of injected dose, p < 0.05). Perfusing hearts with CCCP for a longer period resulted in no greater degree of ^99m^Tc-MIBI washout. The cardiac retention of ^99m^Tc-NOET (42.1 ± 13.4% of ID) was unaffected by CCCP infusion at any concentration examined. The ICR for ^99m^Tc-MIBI progressively increased after 5 minutes of CCCP infusion from 1.27 ± 0.12 to 3.18 ± 0.53 at 600 nM (p < 0.05), while its amplitude did not change (Supplementary Fig. 2). These rates and amplitudes did not change significantly between 5 and 25 minutes of CCCP infusion. The FCR for ^99m^Tc-NOET washout and its amplitude were unaffected by CCCP infusion at any dose at any time point. There was a trend of increasing SCR with increasing CCCP dose, which only reached significance above control values after 25 minutes of infusion with 600 nM CCCP (from 0.004 ± 0.001 to 0.012 ± 0.002 (p < 0.05); the amplitude of the SCR was unchanged with any dose.

#### Doxorubicin perfusion

**Figure 3:**
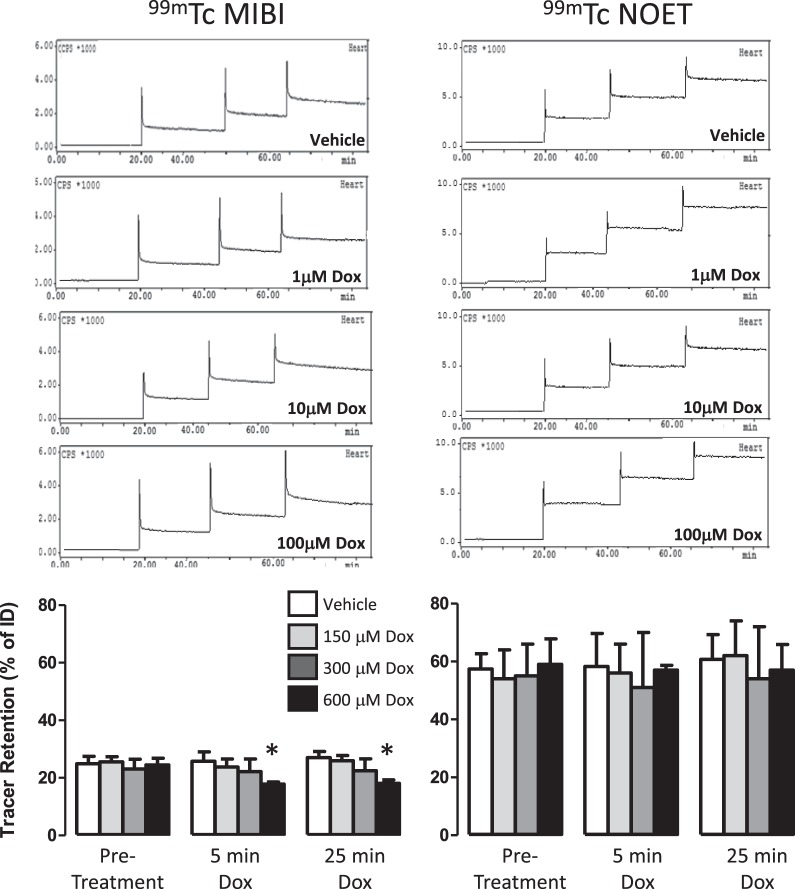
Time-activity curves showing the myocardial clearance/accumulation of ^99m^Tc-MIBI (left) and ^99m^Tc-NOET (right) during perfusion with increasing concentrations of doxorubicin. Graphs represent summarised data of mean (n = 6) ± SD. *Significantly different from pre-intervention control bolus (p < 0.05).

Doxorubicin perfusion caused a dose-dependent loss of cardiac ^99m^Tc-MIBI retention, becoming significantly lower than control values at 100 μM, (17.8 ± 0.7 versus 23 ± 3.5% ID, p < 0.05, Fig. 3). This increased washout was largely described by increased SCR without a change in its amplitude (Supplementary Fig. 3). ^99m^Tc-NOET retention was unaffected by doxorubicin perfusion at any concentration at any time point.

### Langendorff hemodynamics

Hemodynamic parameters are shown in Supplementary Fig. 4. Fifty minutes of hypoxic buffer perfusion invoked an initial hyperemic response which progressed to an elevated perfusion pressure 137% above baseline (p < 0.05), LVEDP increased tenfold, while LVDevP fell to 5% of control. CCCP perfusion caused a dose-dependent increase in perfusion pressure, peaking at 160% above baseline with 600 nM, with a progressive increase in LVEDP to 110.1 ± 6.1 mmHg and fall in LVDevP to 2.7 ± 2.2 mmHg. Doxorubicin perfusion caused a dose-dependent increase in perfusion pressure peaking at 148.7 ± 26.3 mmHg with 100 mM, while LVDevP fell to 29.5 ± 12.9 mmHg.

### The effect of doxorubicin injection *in vivo*

**Figure 4:**
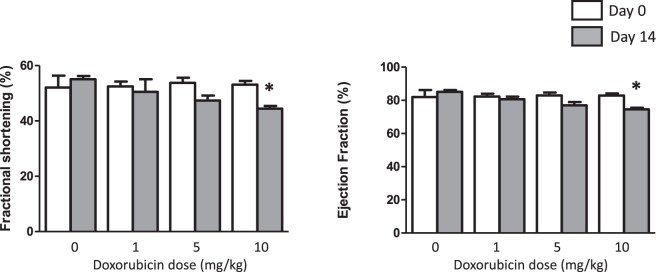
Graphs showing changes in fractional shortening (left) and ejection fraction (right) two weeks after intraperitoneal injection of doxorubicin. Mean ± SD. n = 4–6 *significantly different from pre-intervention control values (p < 0.05).

Intraperitoneal administration of doxorubicin resulted in a dose-dependent thinning of the cardiac septal and posterior ventricular walls 2 weeks later (measured *in vivo* by echocardiography, Table 1). Left ventricular systolic volume progressively increased with increasing doxorubicin dose from 50.3 ± 11.6 to 72.7 ± 9.1 (p < 0.05). Ejection fraction and fractional shortening were only affected at the highest dose of 10 mg/kg (by 7% and 9.5% respectively p < 0.05, Fig. 4). There were no differences in these recorded functional changes between animals assigned to either radiotracer imaging group.

**Table 1:** Changes in Cardiac geometry due to doxorubicin treatment.

	LVIDs (mm)	LVIDd (mm)	IVSs (mm)	IVSd (mm)	LVPWs (mm)	LVPWd (mm)
**Baseline (** ^**99m**^ **Tc-MIBI)**						
0 mg/kg	3.73 ± 0.16	6.56 ± 0.29	2.86 ± 0.15	1.72 ± 0.19	2.40 ± 0.26	1.71 ± 0.25
1 mg/kg	3.76 ± 0.33	6.53 ± 0.37	3.04 ± 0.19	1.94 ± 0.09	2.50 ± 0.34	1.67 ± 0.26
5 mg/kg	3.70 ± 0.51	6.33 ± 0.48	3.13 ± 0.32	1.85 ± 0.10	2.54 ± 0.28	1.74 ± 0.20
10 mg/kg	3.34 ± 0.67	6.20 ± 0.65	2.77 ± 0.20	1.68 ± 0.24	2.53 ± 0.26	1.86 ± 0.13
**Baseline (** ^**99m**^ **Tc-NOET)**						
0 mg/kg	3.44 ± 0.57	6.37 ± 0.65	3.05 ± 0.25	1.75 ± 0.11	2.81 ± 0.27	2.11 ± 0.51
1 mg/kg	3.38 ± 0.32	6.31 ± 0.19	2.93 ± 0.21	1.96 ± 0.23	2.76 ± 0.20	1.86 ± 0.10
5 mg/kg	3.26 ± 0.60	5.93 ± 0.58	3.27 ± 0.25	2.26 ± 0.26	2.54 ± 0.15	1.79 ± 0.14
10 mg/kg	3.16 ± 0.48	6.10 ± 0.27	3.34 ± 0.14	2.08 ± 0.29	2.68 ± 0.12	2.05 ± 0.26
**Week 2 (** ^**99m**^ **Tc-MIBI)**						
0 mg/kg	3.45 ± 0.34	6.10 ± 0.36	2.74 ± 0.03	1.77 ± 0.10	2.56 ± 0.21	2.01 ± 0.22
1 mg/kg	3.77 ± 0.44	6.23 ± 0.14	2.88 ± 0.17	2.04 ± 0.21	2.44 ± 0.23	1.90 ± 0.20
5 mg/kg	**4**.**10 ± 0**.39^*^	6.31 ± 0.57	2.90 ± 0.53	**1**.**58 ± 0**.09^*^	2.47 ± 0.26	1.91 ± 0.24
10 mg/kg	**4**.**31 ± 0**.52^*^	6.76 ± 0.58	**2**.**93 ± 0**.12^*^	**1**.**52 ± 0**.14^*^	2.19 ± 0.33	**1**.**65 ± 0**.11^*^
**Week 2 (** ^**99m**^ **Tc-NOET)**						
0 mg/kg	3.12 ± 0.25	6.02 ± 0.22	2.99 ± 0.25	1.81 ± 0.11	2.68 ± 0.08	2.06 ± 0.26
1 mg/kg	3.47 ± 0.45	6.10 ± 0.44	2.77 ± 0.10	1.81 ± 0.18	2.52 ± 0.18	1.91 ± 0.09
5 mg/kg	3.60 ± 0.79	6.07 ± 0.68	**2**.**55 ± 0**.24^*^	**1**.**79 ± 0**.28^*^	2.18 ± 0.52	1.66 ± 0.25
10 mg/kg	**4**.**11 ± 0**.31^*^	**6**.**96 ± 0**.33^*^	**2**.**68 ± 0**.22^*^	**1**.**72 ± 0**.09^*^	**2**.**33 ± 0**.29^*^	**1**.**73 ± 0**.05^*^

Left ventricular internal dimension at systole (LVIDs); Left ventricular internal dimension at diastole (LVIDd); Intraventricular septum at systole (IVSs); Intraventricular septum at diastole (IVSd); Left ventricular posterior wall at systole (LVPWs); Left ventricular posterior wall at diastole (LVPWd); ^*^P < 0.05 versus baseline value by t-test, n = 4–5.

**Figure 5:**
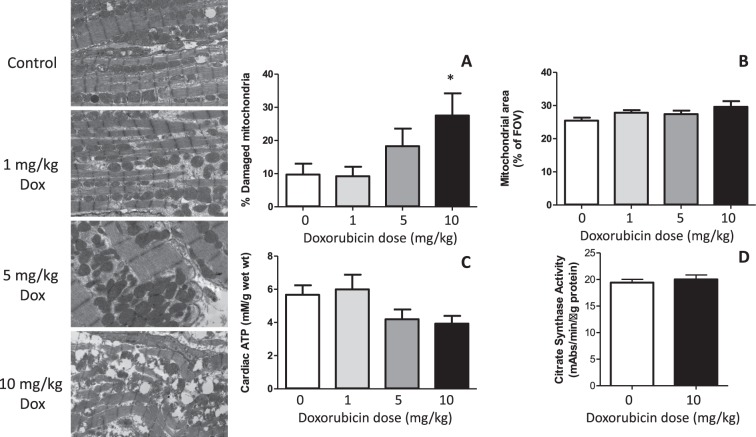
(Left) Electron micrographs showing representative damage in hearts two weeks after administration of each dose of doxorubicin. (**A**) Summarised quantification of % mitochondria damaged and (**B**) average mitochondrial area in the field of view (60 images per heart), (**C**) total cardiac ATP levels and citrate synthase activity in the same hearts. Mean ± SD. n = 4–6. *Significantly different from vehicle controls (p < 0.05).

10 mg/kg doxorubicin administration induced cytoplasmic vacuolisation and myofibrillar disarray (Fig. 5), and a threefold increase in the apparent number of damaged mitochondria per field of view (Fig. 5A). While there was a trend of increased mitochondrial area with increasing doxorubicin dose (Fig. 5B), this did not reach statistical significance; neither was there any quantifiable loss of total mitochondrial content when assessed by citrate synthase activity (Fig. 5D). Doxorubicin administration led to a progressive depletion of cardiac ATP content (Fig. 5C), but this did not reach statistical significance.

**Figure 6:**
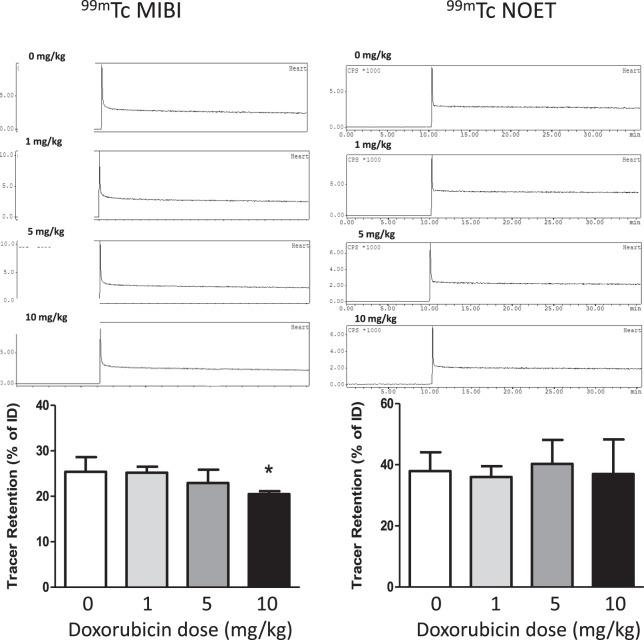
Time-activity curves showing the myocardial clearance/accumulation of ^99m^Tc-MIBI (left) and ^99m^Tc-NOET (right) in hearts excised two weeks after intraperitoneal doxorubicin injection, mean (n = 4–5) ± SD. *Significantly different from vehicle control (p < 0.05).

When hearts from these doxorubicin-treated animals were excised and Langendorff-perfused with normal oxygenated drug-free buffer in the triple γ-detection system, there were no observable differences in their hemodynamic performance (supplemental Fig. 1). However, we observed a dose dependent loss in their capacity to retain ^99m^Tc-MIBI (decreasing from 25.4 ± 3.2% in vehicle controls to 20.5 ± 0.3% in the 10 mg/kg treated group p < 0.05, Fig. 6).

**Figure 7:**
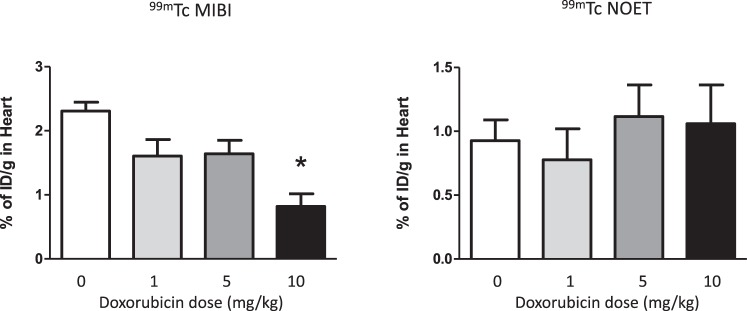
Graphs of cardiac retention of ^99m^Tc-MIBI (left) and ^99m^Tc-NOET (right) injection by biodistribution two weeks after intraperitoneal doxorubicin injection, mean (n = 4–5) ± SD. *Significantly different from vehicle control (p < 0.05).

In parallel groups of animals where cardiac uptake of each tracer *in vivo* was quantified by cut-and-count 3 hours after tail vein injection, there was a marked loss of cardiac ^99m^Tc-MIBI retention with increasing doxorubicin dose (from 2.3 ± 0.3 to 0.9 ± 0.2ID/g with 10 mg/kg doxorubicin, p < 0.05, Fig. 7). Cardiac ^99m^Tc-NOET retention was unaffected by any dose investigated in either study.

## Discussion

We have demonstrated that cardiac ^99m^Tc-MIBI retention is highly sensitive to mitochondrial perturbation induced by hypoxia, mitochondrial uncouplers or doxorubicin treatment in the isolated perfused heart. We then show that sub-acute doxorubicin administration leads to a measurable loss of cardiac ^99m^Tc-MIBI retention *in vivo*, suggesting that it could be used to report on changes in ΔΨ_m_ as a predicator of evolving cardiac injury. For this potential to be realized, it would be necessary to correct clinical cardiac ^99m^Tc-MIBI images for the regional variations in perfusion which arise naturally or through concomitant cardiovascular disease or injury. To this end, we demonstrate for the first time that the cardiac retention and pharmacokinetics of the perfusion tracer ^99m^Tc-NOET, an uncharged lipophilic complex, are insensitive to the perturbations that affect ^99m^Tc-MIBI, suggesting that it would be suitable for this purpose.

While the cardiac biodistribution of ^99m^Tc-MIBI is used extensively clinically to delineate myocardial perfusion, early isolated cell work demonstrated that 90% of its cardiac accumulation is trapped within mitochondria according to ΔΨ_m_, and is blockable by metabolic inhibitors like rotenone and ionophores such as FCCP^20,29^, or promoted by mitochondrial hyperpolarization using nigericin^30^. To determine how ^99m^Tc-MIBI might best be used clinically for detecting the development of anthracycline cardiotoxicity, it is essential to characterize the kinetics of the tracers in intact myocardium, as reported here. We have demonstrated that cardiac ^99m^Tc-MIBI washout increased with hypoxia severity and duration in isolated perfused hearts, and showed a dose-dependent loss of cardiac ^99m^Tc-MIBI retention with increasing dose of the ionophore CCCP and doxorubicin. Our results are comparable to studies investigating mitochondrial trapping of the lipophilic cation TMRE in FCCP-treated isolated hearts using surface fluorescence^31,32^. Our radiometric signal, however, is not subject to the complications of auto-fluorescence and quenching common in fluorescence measurement, or the poor tissue penetration which limits the translational potential of optical imaging techniques.

Progressing to rats *in vivo*, we confirm that we can detect loss of ^99m^Tc-MIBI retention indicative of cardiotoxicity two weeks after a single i.p. bolus of doxorubicin. This is unlikely to be a direct ^99m^Tc-MIBI-doxorubicin interaction (since doxorubicin has a half-life of 17.9 and 17.7 hours in plasma and cardiac tissue respectively^33^), but rather the mitochondrial perturbation caused by the initial doxorubicin insult. While mitochondrial disarray was evident by electron microscopy at the highest dose of 10 mg/kg, there was no measurable loss of cardiac citrate synthase activity at any dose used, suggesting that ^99m^Tc-MIBI washout was not caused by loss of mitochondrial number. There was a modest loss of cardiac ATP which did not reach statistical significance even at the top dose of doxorubicin used, highlighting the ability to detect subtle mitochondrial dysfunction which precedes or is at a level below that which can be measured by existing functional/biophysical techniques. We demonstrate loss of cardiac ^99m^Tc-MIBI retention with all doxorubicin dosages used, which although only reaching statistical significance at the highest dose of 10 mg/kg in this small proof of concept study, we believe is attributable to the variability in cardiotoxic injury induced by a single intraperitoneal injection, rather than variability in our ability to measure it. In our ongoing work we are refining our doxorubicin administration methodology to improve its reproducibility, but this does underline the clinical problem: it is difficult to predict which individuals will exhibit cardiotoxicity following anthracycline administration (and to what degree), even when an identical dosing regime is used. Personalized predictors of evolving cardiac injury in response to anthracyclines are essential.

The dynamic range of ^99m^Tc-MIBI washout in response to doxorubicin was greater when measured by organ counting after *in vivo* injection than when measured using the triple γ-detection system. There are two possible reasons for this: first, the isolated heart experiments have rapid first pass perfusion (100 μl into a coronary flow of 14 ml/min), with no tracer recirculation. As such, the time available for tracer to enter myocytes, transit to mitochondria and equilibrate according to ΔΨ_m_ is very short, meaning that differential uptake between mitochondria at different energy states may be limited by tracer kinetics. *In vivo*, where coronary flow is slower (~3 ml/min), and the tracer bolus is likely more prolonged (with the opportunity for second pass uptake as the tracer recirculates), there may be greater tracer uptake and equilibration according to ΔΨ_m_, providing a greater dynamic range of measurement. Second, ^99m^Tc-MIBI is a known substrate of P-glycoprotein (PgP), which plays a role in pumping the tracer from the cell^34^. Cardiac PgP levels have recently been shown to be upregulated by anthracycline treatment^35^, which may represent a secondary anthracycline-dependent mechanism removing ^99m^Tc-MIBI from the heart. As there was 3 hours between tracer injection and post-mortem biodistribution, this effect may have a greater effect on cardiac ^99m^Tc-MIBI retention when measured *in vivo* compared to the isolated heart. The role of PgP in cardiac ^99m^Tc-MIBI washout *in vivo* warrants further investigation in this context.

Accelerated cardiac ^99m^Tc-MIBI washout kinetics have been reported in patients with dilated cardiomyopathy and hypertrophic myopathy^36^, suggesting that this approach may be sufficiently sensitive to detect cardiac mitochondrial dysfunction clinically. A small (16 patient/group) retrospective clinical trial has explored the potential of using ^99m^Tc-MIBI to detect chemotherapeutic cardiotoxicity. The authors reported higher cardiac ^99m^Tc-MIBI retention in cancer patients post chemotherapy than in their control group, suggesting that chemotherapy led to mitochondrial hyperpolarization^37^ (in contrast to the historic literature cited above, and our current data). However, since the “control” group used in this study were patients undergoing assessment for suspected ischaemic heart disease (rather than healthy controls), it is likely that their hearts were more compromised than the chemotherapy treatment group itself, which may explain the disparity. The author also notes several chemotherapy patients with fixed (12%) and reversible (44%) perfusion defects in his cohort, which would likely impact on the sensitivity of the approach to detect mitochondrial dysfunction, underlining the potential importance of perfusion correction in the interpretation of ^99m^Tc-MIBI data used for this purpose.

To ameliorate this common problem of coexisting perfusion defects, we propose that the sensitivity and specificity of the approach in general could be enhanced by performing sequential or simultaneous scans for mitochondrial perturbation and perfusion. ^99m^Tc-NOET is a neutral lipophilic complex designed for cardiac perfusion imaging based on its lipophilic retention in cell membranes^38,39^. Subcellular biodistribution studies have revealed that it can diffuse and localize into hydrophobic components of myocardial cells (but not mitochondria), suggesting that once taken into the cell, it becomes trapped within intracellular liposomes^39^. *In vivo* studies in rats, dogs and monkeys have demonstrated that it is rapidly extracted by the myocardium proportional to regional blood flow^21,40^. Our data go on to demonstrate that ^99m^Tc-NOET is remarkably insensitive to all of the interventions to which ^99m^Tc-MIBI responds (hypoxia, ΔΨ_m_ collapse, and doxorubicin administration *ex vivo* and *in vivo*). Furthermore, since we observed no change in cardiac ^99m^Tc-NOET retention in any animals treated with doxorubicin at any dose, it confirms that the observed changes in ^99m^Tc-MIBI retention were not caused by decreased tracer delivery through loss of perfusion. As such, ^99m^Tc-NOET is well suited to being used as a comparative agent for correcting for regional variations in perfusion to unmask mitochondrially-dependent loss of ^99m^Tc-MIBI retention.

We propose ^99m^Tc-MIBI and ^99m^Tc-NOET as an initial proof-of-principle pairing due to the widespread clinical use of ^99m^Tc-MIBI. However, there is scope for refinement to our approach. Dual isotope imaging could allow ΔΨ_m_ and perfusion measurements to be performed in a single scanning session by co-injecting an uncharged lipophilic equivalent of NOET radiolabelled with a different gamma-emitting radioisotope, offering advantages in spatiotemporal co-registration of scanning and tracer kinetic information. Our initial curve fitting data in the isolated heart suggest a 17-fold increase in the measured SCR between patent mitochondria and those depolarized by hypoxia, suggesting the potential for parameter mapping. The FCR appears insensitive to most interventions we studied, and is likely to largely reflect perfusion^24^. It could therefore possibly be exploited to correct the SCR in a single dynamic scan, but this would require further validation and characterization *in vivo*. While quantifying differences between early and late images as a measurement of ^99m^Tc-MIBI washout may be sufficient to crudely estimate tracer washout rate, for high temporal resolution measurement of kinetics, SPECT is limited because of the time required for the gantry to rotate. This could be mitigated by using dynamic planar scintigraphy at the expense of three-dimensional information. However, translation of the approach to PET would provide advantages in sensitivity, spatial resolution, and the native capacity for 3D dynamic imaging and pharmacokinetic modeling. We and others are currently developing lipophilic cationic PET imaging agents (and lipophilic perfusion imaging agents as comparators) with sufficiently short half-lives to allow sequential quantification of perfusion mitochondrial function in a single scan session; rapidly evolving PET/MR technologies may also provide opportunities for simultaneous MR perfusion correction of PET data.

## Electronic supplementary material

Dataset 1

## Data Availability

The datasets generated during and/or analysed during the current study are available from the corresponding author on reasonable request.
