# Supplementary material for: Detection of anthracycline-induced cardiotoxicity using perfusion-corrected 99mTc sestamibi SPECT
Source: Sci Rep. 2019 Jan 18;9:216. doi: 10.1038/s41598-018-36721-5 (PMC6338786; doi:10.1038/s41598-018-36721-5)
Supplement: Supplementary file 1 — Dataset 1 [file 41598_2018_36721_MOESM1_ESM.docx]

**Supplemental figures.**

Detection of anthracycline-induced cardiotoxicity using perfusion-corrected ^99m^Tc sestamibi SPECT

Zaitulhusna M Safee Ph.D., Friedrich Baark M.Sc., Edward CT Waters M.Sc., Mattia Veronese Ph.D., Victoria R Pell Ph.D, James E Clark Ph.D., Filipa Mota Ph.D., Lefteris Livieratos Ph.D. , Thomas R Eykyn Ph.D., Philip J Blower D.Phil., Richard Southworth Ph.D.

Supplemental Figure 1


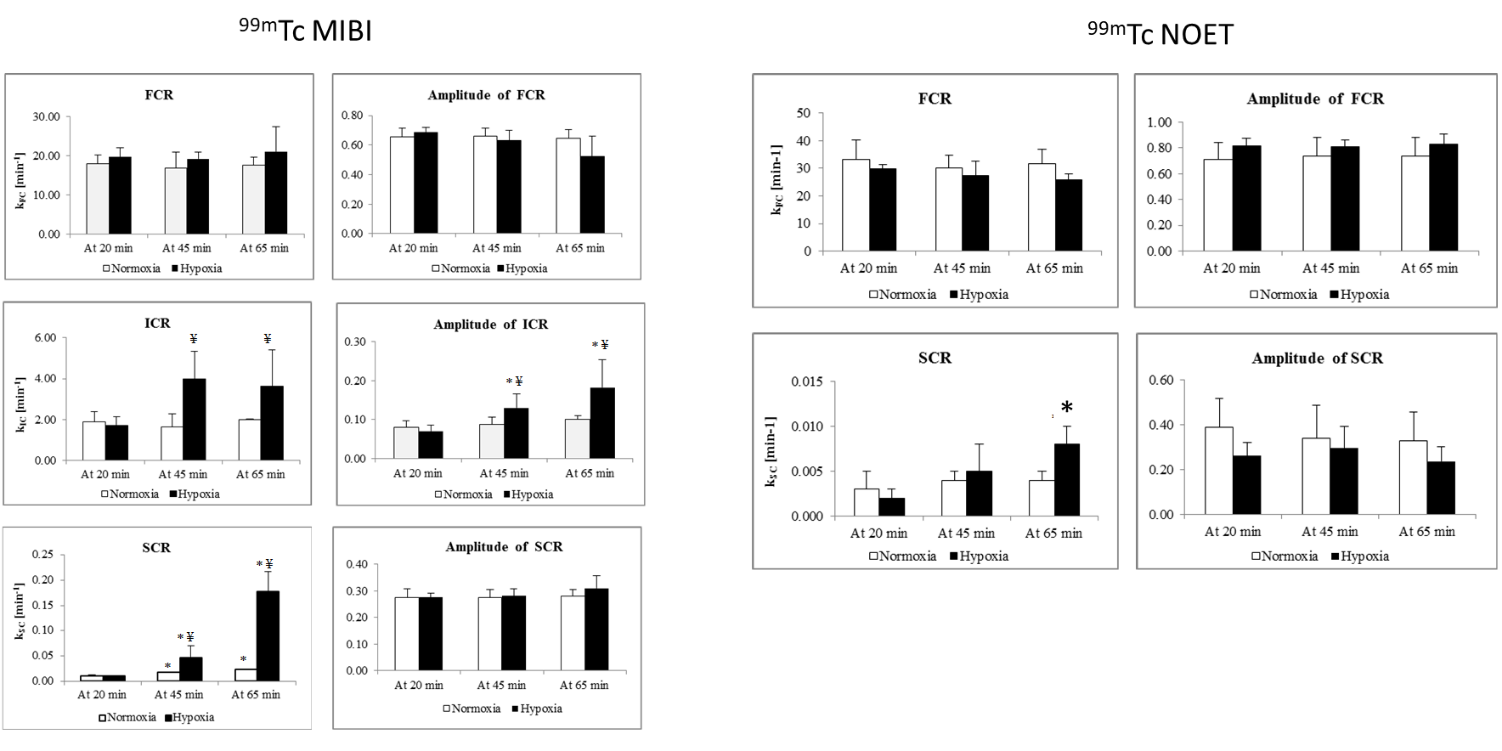


Supplemental figure 1. Graphs showing changes in the respective rates of washout of ^99m^Tc-MIBI (left) and ^99m^Tc-NOET (right) and their amplitudes during normal aerobic perfusion (white bars) and hypoxic buffer perfusion (black bars). FCR, ICR and SCR represent the fast, intermediate and slow clearance rates respectively. mean (n=4-5/group) ± SD. * = significantly different from vehicle control (p<0.05).

Supplemental Figure 2


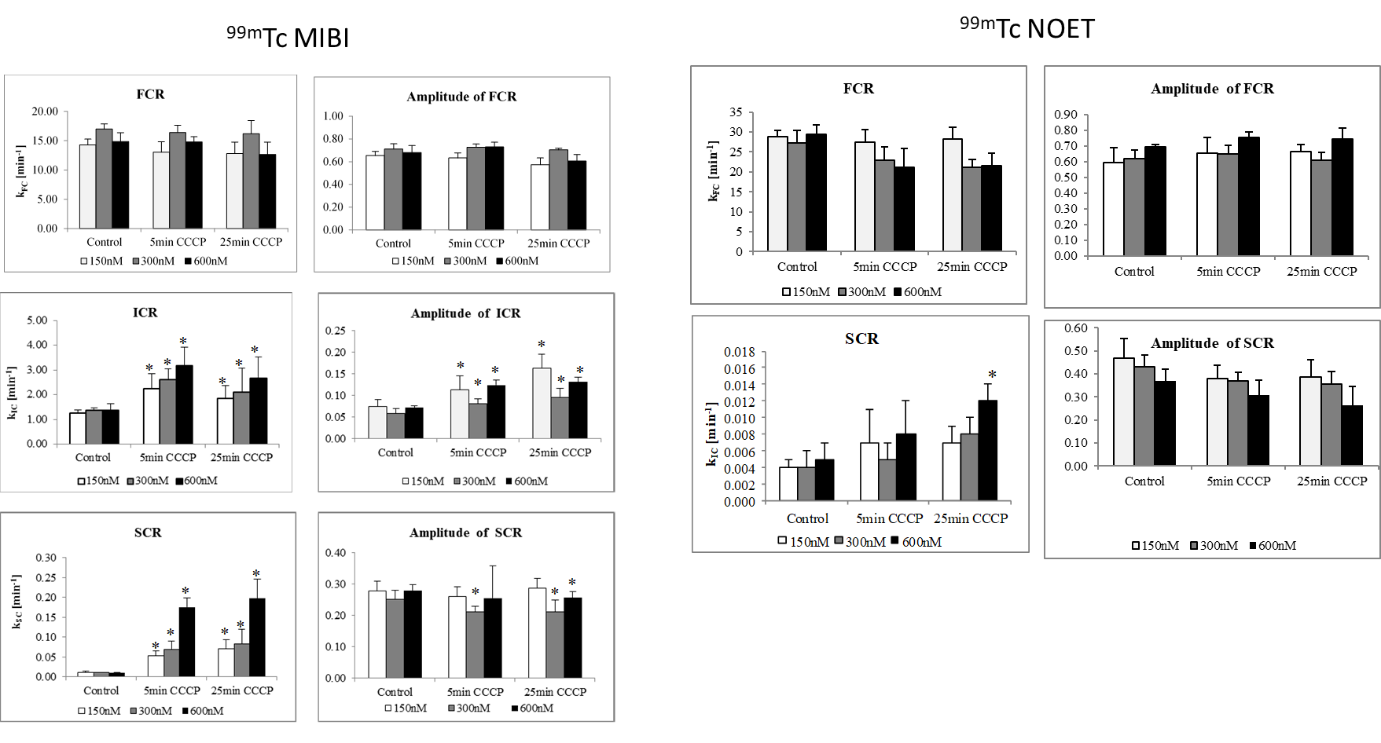


Supplemental figure 2. Graphs showing changes in the respective rates of ^99m^Tc-MIBI (left) and ^99m^Tc-NOET (right) and their amplitudes either before (control), 5 or 25 minutes after perfusion with the ionophore CCCP at either 150 μM (white bars), 300 μM (grey bars) or 600 μM (black bars). FCR, ICR and SCR represent the fast, intermediate and slow clearance rates respectively, mean (n=4-5/group) ± SD. * = significantly different from vehicle control (p<0.05).

Supplemental Figure 3


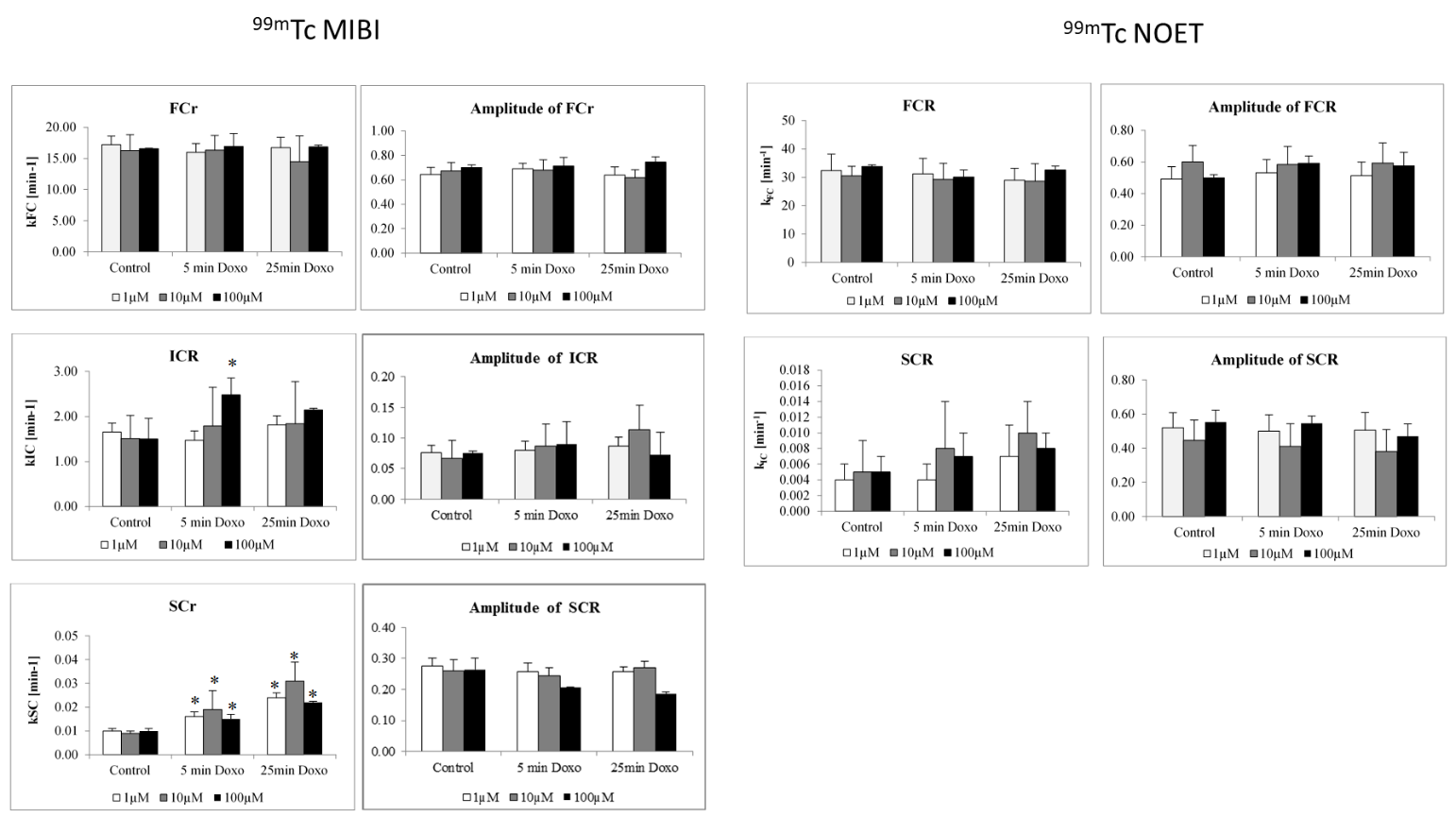


Supplemental figure 3. Graphs showing changes in the respective rates of ^99m^Tc-MIBI (left) and ^99m^Tc-NOET (right) and their amplitudes either before (control), 5 or 25 minutes after perfusion with the doxorubicin at either 1 μM (white bars), 10 μM (grey bars) or 100 μM (black bars). FCR, ICR and SCR represent the fast, intermediate and slow clearance rates respectively, mean (n=4-5/group) ± SD. * = significantly different from vehicle control (p<0.05).

Supplemental Figure 4


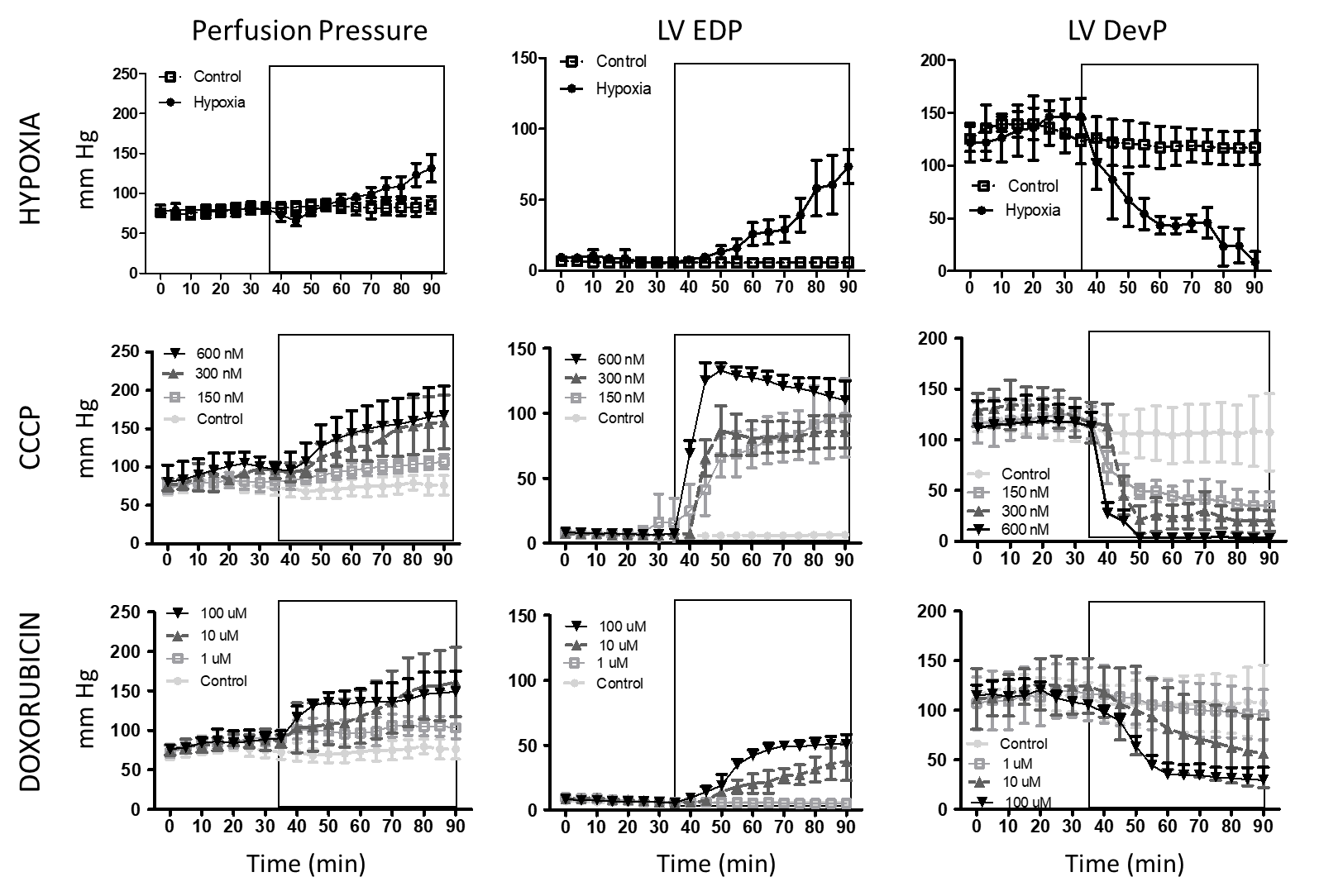


Supplemental figure 4. Graphs showing haemodynamic response in isolated perfused hearts in response to perfusion with hypoxic buffer (top row), perfusion with increasing concentrations of the ionophore CCCP (middle row), or doxorubicin (bottom row). Data shown are coronary perfusion pressure (left column), left ventricular end diastolic pressure (LV EDP, middle column), and left ventricular developed pressure (right column) (n=8-10/group) ± SD. * = significantly different from vehicle control (p<0.05).
